# CancerPPD2: an updated repository of anticancer peptides and proteins

**DOI:** 10.1093/database/baaf030

**Published:** 2025-05-07

**Authors:** Milind Chauhan, Amisha Gupta, Ritu Tomer, Gajendra P S Raghava

**Affiliations:** Department of Computational Biology, Indraprastha Institute of Information Technology, Okhla Phase 3, New Delhi 110020, India; Department of Computational Biology, Indraprastha Institute of Information Technology, Okhla Phase 3, New Delhi 110020, India; Department of Computational Biology, Indraprastha Institute of Information Technology, Okhla Phase 3, New Delhi 110020, India; Department of Computational Biology, Indraprastha Institute of Information Technology, Okhla Phase 3, New Delhi 110020, India

## Abstract

CancerPPD2 (http://webs.iiitd.edu.in/raghava/cancerppd2/) is an updated version of CancerPPD, developed to maintain comprehensive information about anticancer peptides and proteins. It contains 6521 entries, each entry provides detailed information about an anticancer peptide/protein that include origin of the peptide, cancer cell line, type of cancer, peptide sequence, and structure. These anticancer peptides have been tested against 392 types of cancer cell lines and 28 types of cancer-associated tissues. In addition to natural anticancer peptides, CancerPPD2 contains 781 entries for chemically modified and 3018 entries for N-/C- terminus modified anticancer peptides. Few entries are also linked with 47 clinical studies and have provided the cross reference to Uniprot, DrugBank, and ThPDB2. The possible entries also linked with clinical trials. On average, CancerPPD2 contains around 85% more information than its previous version, CancerPPD. The structures of these anticancer peptides and proteins were either obtained from the Protein Data Bank (PDB) or predicted using PEPstrMOD, I-TASSER, and AlphaFold. A wide range of tools have been integrated into CancerPPD2 for data retrieval and similarity searches. Additionally, we integrated a REST API into this repository to facilitate automatic data retrieval via program.

**Database URL**: https://webs.iiitd.edu.in/raghava/cancerppd2/api/rest.html

## Introduction

Cancer is a potentially lethal disorder characterized by significant molecular and genetic alterations that lead to uncontrolled cell proliferation and multiplication, resulting in tissue mass enlargement in the affected area. According to the International Agency for Research on Cancer (IARC), there were an estimated 20 million new cancer cases and 9.7 million deaths in 2022. The most effective cancer treatment procedures currently in use are chemotherapy, radiation, surgery, or their combinations. Despite tremendous efforts over the years, these traditional treatments have achieved limited success due to a number of reasons including acquired drug resistance against chemotherapy [1]. Researchers are now exploring alternative strategies for treating cancer patients, with anticancer peptides and proteins emerging as a promising alternative to traditional cancer therapies [2, 3]. These peptides have a number of advantages over small molecules that include deep tissue penetration, efficient internalization into cells, lower toxicity to tissues [1, 4–6].

In 2015, we compiled experimentally validated anticancer peptides and proteins from literature and public domain repositories to aid the scientific community. To the best of our knowledge, CancerPPD is the only database that provides complete and comprehensive information on anticancer peptides [7]. Over the past decade, this database has been extensively used and cited in scientific research. Since the inception of CancerPPD, the number of methods developed for predicting and designing anticancer peptides has grown exponentially. Most existing anticancer peptide prediction methods have derived datasets from CancerPPD to build their models, such as AntiCP, ACP-DRL, MA-PEP, ACP-ML, ACPPfel, AntiCP2, MLACP 2.0, and ACP-check [8–14]. This highlights the significance of CancerPPD in cancer biology, particularly in the design of peptides and proteins for treating cancer patients.

In the past decade, numerous anticancer peptides have been discovered and experimentally validated by researchers. While these peptides are available in literature and public domain databases, they are not accessible from a single source. To address this problem, we updated our repository, CancerPPD, to consolidate comprehensive information about these peptides. This paper describes the information maintained in our updated repository, CancerPPD2.

## Materials and methods

### Data collection and compilation

We searched NCBI PubMed, Google Scholar, and Patent Lens from October 2014 to March 2024 for anticancer peptide and protein information. We searched for ‘ACPs’, ‘antitumor peptides’, ‘anti-angiogenic’, ‘anti-metastatic’, and ‘host defense peptides’ to get more information. We also searched UniProt, PDB, PubChem, and ChEMBL for peptide and protein sequences, structures, and SMILES. Tabular data from numerous sources was added to the CancerPPD 2.0 database and unnecessary information was eliminated. We thoroughly checked all data before adding it to our database to ensure quality.

### Database architecture and web interface development

**Figure 1.**
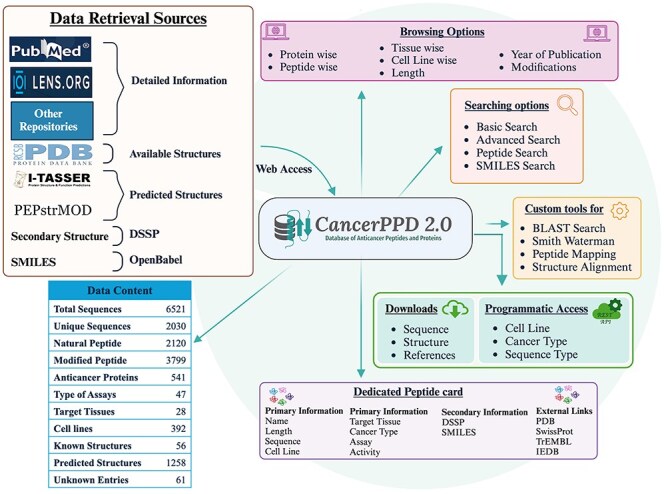
The figure shows the complete architecture of the database.

The database was created on the Linux platform using the Apache HTTP server, with all back-end data managed by MySQL. It provides instructions on how to extract and store data. The front-end web interface was designed and enhanced using JavaScript, HTML, CSS, and Bootstrap framework. Scripts for the database interface and common gateway interface were written in PHP. The web interface is completely responsive allowing users to access our database from a wide range of devices including smart devices. The Fig. 1 given below shows the complete architecture of the database.

### Data content

CancerPPD-2.0 is a manually curated database of anticancer proteins and peptides’ sequence, length, experimental technique, structure, and SMILES. The primary data include the peptide name, sequence, length, configuration, chirality, chemical modification, origin, anticancer activity, tested cell lines, assay and cancer kinds, target tissues, and PMID. Secondary information, like tertiary structures and SMILES, is based on primary information. We compiled all peptide structures and SMILES for an exhaustive database. Firstly, we mined the Protein Data Bank (PDB) to collect existing peptide/protein structures. Secondly, we used PEPstrMOD, an updated version of PepStr for predicting structure of peptides [15, 16]. To the best of our knowledge, PEPstrMOD is the only method that can predict the structure of peptides containing natural or chemically modified peptides. Thus, all peptides in our dataset were predicted using PEPstrMOD whether they are natural peptides or chemically modified or N-/C-terminal-modified peptides. In case peptides contain more than 25 residues, we expanded PEPstrMOD to 40 residues to include more natural and modified peptides. The structure of anticancer peptides/proteins obtained from UniProt was extracted from the cross-reference database ‘AlphaFoldDB’. For anticancer proteins whose structure is not available in ‘AlphaFoldDB’ was predicted using software I-TASSER [17, 18].

## Implementation of tools

### Data retrieval

Number of modules has been integrated inthe web server to perform data search in the database. One of the simple modules for performing standard search is the ‘Basic Search’ module. This module simplifies search with multiple options. One query at a time can be searched using PMID, year of publication, peptide sequence or name, peptide characteristics, cell line or origin, etc. Users can also pick several search phrases. Select fields will appear on screen. One may perform complex searches in CancerPPD2 using the ‘Advanced Search’ module. Its multiple query system lets users input various keywords with single or multiple search queries. Allow to use standard operators like ‘AND’ and ‘OR’ to perform conditional searches for multiple queries. The ‘Peptide Sequence’ option lets users compare a peptide sequence to all peptide sequences of CancerPPD2.0. It has the option to perform identical or subsequence search, where identical searches extract identical peptides from the database. Subsequence searches retrieve amino acid-containing peptide sequences from the query peptide. SMILES converts protein or peptide 3D structures into symbols. The ‘SMILES Search’ module allows atom-level peptide sequence searches. ACPs can be searched for atoms, bonds, and groups. CancerPPD2.0 users can search SMILES notation of a query peptide by substructure, exact, exact fragment, or superstructure.

### Browsing tools

The browsing tool organizes content for easy access. The browsing tool lists numerous ACP-identification submodules. Submodules include ‘Protein wise’, ‘Peptide wise’, ‘Tissue wise’, ‘Cell Line wise’, ‘Assay type’, ‘Length wise’, ‘Modification wise’, ‘Clinical Trials’ ‘Publication year’, and ‘Uniprot Proteins’ (Uniprot-sourced proteins). The database contained 392 cancer cell line types from 28 tissue types. Users can browse ACPs tested against various cancer cell lines. The peptide card for each entry contains detailed information about a peptide or protein. Key information on the peptide card includes PMID, sequence, length, origin, etc. This material covers chemical, C-terminal, and N-terminal changes. Activity, test, cell line, and clinical studies data are also included. Literature information includes paper title, DOI, journal, and abstract. It also includes secondary information like DSSP and SMILES. External connections to PDB, SwissProt, and TrEMBL are also provided. CancerPPD 2.0 includes experimental and predicted peptide and protein structures from the PDB. The NGL viewer is used to display these structures [19].

### Analysis tools

We integrated several online tools to compare molecular structures and seek for sequence similarities. The basic local alignment search tool (BLAST) used to compare query sequences with closely related ACP sequences included in the database. We created a Smith-Waterman-based similarity search engine to improve sequence similarity analysis. Users can find ACPs in their proteins using the peptide-mapping tool. After the user enters a protein query, the server identifies and records the ACPs. We also offer a structural alignment method that lets users align structures using a PDB file with a CancerPPD2.0 ID.

### Download

The complete CancerPPD 2.0 data are available for download. Downloading a multifasta file provides unaltered and altered sequences. PDB files include predicted and mapped PDB structures. We also let users download or observe search query results in CSV or Excel format. Download all PubMed open-access reference publications.

### An API

We added programmatic data retrieval from CancerPPD 2.0 in this upgraded release to reduce manual downloads. Programs can use simple URLs (REST) to retrieve data by cell line, cancer type, and sequence type. The API returns data in JSON format, a standard data exchange format. Users can customize JSON parsing.

## Result and discussion

The updated version of CancerPPD, CancerPPD2.0, holds a total of 6521 entries with every available information we possibly retrieve from the literature and Uniprot. Out of which, 5919 entries include information of anti-cancer peptides, 541 anticancer proteins and 61 Unknown entries. The Unknown category had missing sequence information such as amino acid sequence and/or length while we retrieved other information about them such as their anticancer activity, cell lines on which they were tested, assays, tissue types and so on [20–22]. Given the varied activity type of ACPs on different cell lines, we added multiple entries for a single ACP if their cell lines, IC50 values, assays, or tissue types differ. We have included data for 47 clinical trial entries linked with NCT IDs, 392 cancer cell lines corresponding to 28 tissue types. Additionally, we have updated the number of chemically modified entries to 781, as the stability of peptides is a major concern in developing therapeutic peptides.

In the updated version, we have a total of 2661 entries for 1005 natural anticancer peptides and proteins. Out of 1005 ACPs, we obtained structures for 111 ACPs from PDB as their structures are available in PDB. We predicted the structure of 541, 401, and 52 ACPs using PEPstrMOD, AlphaFold, and I-TASSER, respectively. We were unable to predict the structure of peptides which have less than five amino acids. Our database has 3018 entries for 742 ACPs, whose N- or C-terminals are modified. We predicted the structure of 673 out of 742 ACPs using PEPstrMOD. Our database also has 781 entries for 251 ACPs which have chemical modification other than N- or C-terminal. Out of 251 chemically modified, we were able to predict the structure of only 33 chemically ACPs using PEPstrMOD. We failed to predict the structure of a number of chemically modified ACPs due to unavailability of forcefield in PEPstrMOD. These modified structures of peptides/proteins involved complex chemical modifications like to 1-amino-isobutyric acid, beta-naphthylalanine, norleucine, Ornithine etc. Along with the complex modifications, some of the structures were also not predicted due to lack of sequences as their complex structures were given as figures in the source publications [23–26].

**Figure 2.**
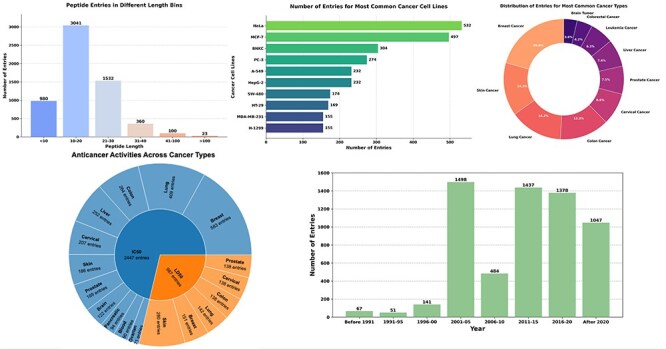
The figure shows the diversity and distribution of anticancer peptides.

The Fig. 2 provides a detailed visualization of the CancerPPD 2.0 database, showcasing the diversity and distribution of anticancer peptides. The top left bar chart illustrates the number of peptide entries across different length bins. The adjacent donut chart displays the distribution of entries among the 10 most common cancer types. The lower left bar chart presents the number of entries for the 10 most frequently studied cancer cell lines. The adjacent bar chart shows the number of peptide entries across various year bins. The given pie chart shows the anticancer activity of peptides/proteins with cancer type.

The ACPs are found to be cationic, amphipathic, and hydrophobic in nature [27]. They also have high efficiency in tissue penetration and cell internalization [4, 28]. The outer membrane of cancer cells is more anionic as compared to healthy cells, which helps the ACPs attachment to cancer cells and disrupting them either by necrosis or apoptosis [29]. The ACPs are also immunogenic which make their usage suitable for therapeutic purposes [30]. There is a continuous growth has been observed on peptide based research as a number of peptides are currently undergoing different clinical phases [31, 32]. By considering the growing demands of ACPs as therapeutic targets, we updated this repository to help the scientific community to better aid with this serious situation.

## Comparison with the previous version

CancerPPD2.0 is an upgraded version of a thoroughly maintained repository of proteins/peptides with anticancer properties. The initial version of CancerPPD was developed in 2014. Subsequently, a substantial volume of ACP data has been made available. In the revised edition, we have made significant modifications to the ACP data, adding 2612 additional entries from literature (Data mined from Pubmed and Patent Lens) along with their associated primary and secondary information. The updated version includes 491 modified entries, whereas prior versions only had 290 modified entries. It also contains clinical studies linked with their NCT IDs and has provided the cross reference to Uniprot, DrugBank, and ThPDB2. We have also retrieved the protein/peptides from UniProt which are categorized as ‘Anticancer’. Below is a concise comparison between the entries in the prior and revised versions (please refer Table 1). We are of the opinion that this revision greatly improves the data content.

**Table 1.** Comparison of older version of CancerPPD [7] with newer version of CancerPPD 2.0

Keyword	CancerPPD	CancerPPD 2.0	Total Entries
Anticancer peptides	3438	2481	5919
Anticancer proteins	121	420	541
Cell lines	249	226	392
Assays	16	41	49
Modified peptide entries	290	491	781
L	3274	2373	5647
D	26	31	57
Mix	178	183	361
Tissue type	21	24	28
Unique entries (Peptides)	600	939	1539

## Conclusion

CancerPPD2.0 contains 6521 entries and offers improved coverage of all the ACPs. We have updated the data by integrating recently identified ACPs from the most recent studies into the database. The data can be easily accessed either by directly downloading it from the website or by programmatically accessing it. We believe that the revised edition will prove beneficial to the scientific community.

### Limitation and update

In CancerPPD2.0, we have enhanced the database by including around 85% of the information on ACPs. In addition, we have streamlined the process of accessing data by incorporating a REST API allowing programmatic access in conjunction with the web interface. Nevertheless, our current limitations prevent us from accurately forecasting the tertiary structure of the most complex modified peptides such as Methoxylation, Biotinylated, Hydroxamic acid, etc. in this version. This is mostly due to the absence of force field libraries specifically designed to handle such alterations. We intend to rectify this constraint in forthcoming versions, if feasible.

## Supplementary Material

baaf030_Supp

## Data Availability

The database CancerPPD2.0 is freely accessible at ‘https://webs.iiitd.edu.in/raghava/cancerppd2/’ and data can be downloaded from the download tab given in the website or directly from API at https://webs.iiitd.edu.in/raghava/cancerppd2/api/rest.html by selecting the query fields.
