# Supplementary figures and images for: CancerPPD2: an updated repository of anticancer peptides and proteins
Source: Database (Oxford). 2025 May 7;2025:baaf030. doi: 10.1093/database/baaf030 (PMC12060709; doi:10.1093/database/baaf030)

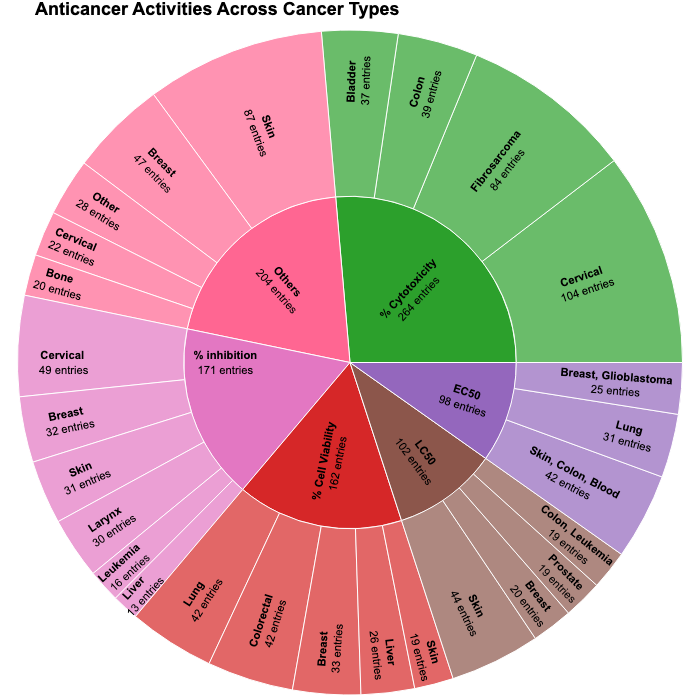

Supplement: baaf030_Supp [file baaf030_supp.zip › Supplementary Figures/figure2_supp.tiff]

**Figure S1: The figure shows the anticancer activity across cancer types.**


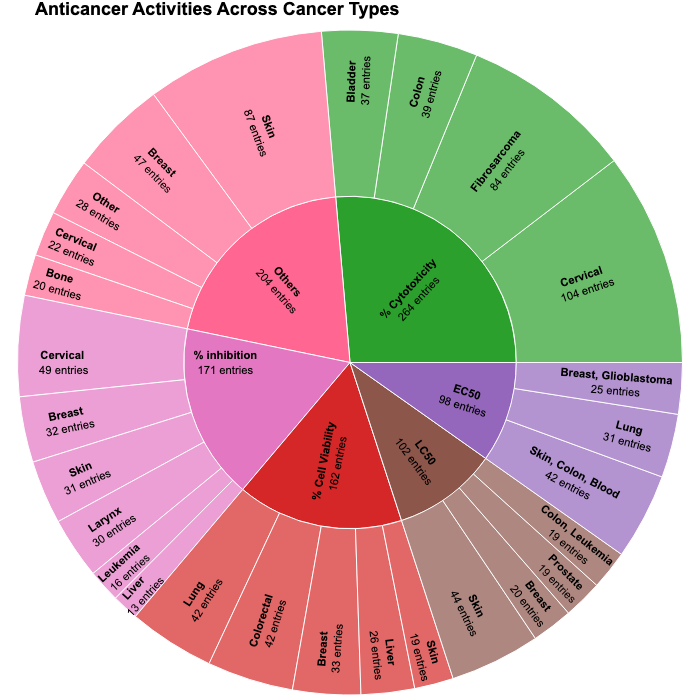

Supplement: baaf030_Supp [file baaf030_supp.zip › Supplementary Figures/Supplementary_figureS1.docx]
